# Modified solid in oil nanodispersion containing vemurafenib-lipid complex- *in vitro*/ *in vivo* study

**DOI:** 10.12688/f1000research.123041.2

**Published:** 2022-09-07

**Authors:** Yasir Q. Almajidi, Nidhal K. Maraie, Ayad M. R. Raauf

**Affiliations:** 1 Department of Pharmaceutics, College of Pharmacy, Mustansiriyah University, Baghdad, Iraq, Iraq

**Keywords:** ConQuest, DLC, Mercury, Phosphatidylethanolamine, SON, Vemurafenib

## Abstract

**Background: **Vemurafenib (VEM) was a licensed drug for the treatment of skin melanoma and is available only in the market as oral tablets prescribed in huge doses (1920 mg/day). One reason for the high dose is vemurafenib's low oral bioavailability.

**Methods: **VEM-lipid complex (DLC) was predicted based on Conquest and Mercury programs and prepared using the solvent evaporation method using the lipid (phosphatidylethanolamine). DLC was subjected to characterization (FT-IR, Raman spectroscopy, DSC, TGA, P-XRD, and FESEM) to confirm complexation.  DLC was used to prepare solid in oil nanodispersion (DLC-SON) and subjected to in vitro, ex vivo, and in vivo evaluation in comparison to our recently prepared conventional SON (VEM-SON) and DLC-control.

**Results: **Conquest and Mercury predict the availability of intermolecular hydrogen bonding between VEM and phosphatidylethanolamine (PE). All characterization tests of DLC ensure the complexation of the drug with PE. Ex vivo studies showed that the drug in DLC-SON has significantly (P<0.05) higher skin permeation than DLC-control but lower drug permeation than conventional SON but it has a higher % skin deposition (P<0.05) than others. The half-maximal inhibitory concentration (IC50) of the prepared DLC-SON is significantly high (P<0.05) in comparison to the conventional SON and pure VEM. In vivo permeation using confocal laser scanning microscopy (on the rat) results indicated that both conventional SON and DLC-SON can cross the SC and infiltrate the dermis and epidermis but DLC-SON has a higher luminance/gray value after 24 h in the dermis in comparison to the conventional SON.

**Conclusion:** The novel lipid complex for VEM prepared using PE as a lipid and enclosed in SON showed higher anticancer activity and topical permeation as well as sustained delivery and good retention time in the dermis that localize the drug in a sufficient concentration to eliminate early diagnosed skin melanoma.

## Introduction

Metastatic melanoma patients have a bad prognosis and only a limited number of therapy alternatives are available. ^1^ Therefore, discovering oncogenic BRAF in most melanomas (80% cases) and showing that BRAF mutant melanoma cells rely heavily on BRAF/MAPK signaling were significant findings with substantial pharmacological impact. Thus, specific BRAF ^V600E^ inhibitors like Vemurafenib (VEM) benefit many melanoma patients. ^2^

VEM poses a substantial difficulty for oral delivery due to its physicochemical properties. In a physiologically relevant pH range, crystalline Vemurafenib has an aqueous solubility of 0.1 g/mL with no pH dependence. in vitro studies, however, revealed that VEM had lower permeability in a Caco-2 intestinal epithelial cells model when compared to a low permeability reference ranitidine. ^3^ VEM is available in the market as a 240 mg oral tablet with a dose of 1920 mg/day (4 tablets twice daily) to treat skin melanoma, VEM accumulated significantly after repeated dosing at 960 mg BID, had a high cost with a high degree of inter-patient variability. One reason for the high dose is VEM’s low oral bioavailability due to low solubility of VEM at physiological pH. There is no marketed IV injection for the drug. ^4^

When the drug’s molecular weight exceeds 500 Da, however, the drug’s permeability through the skin decreases. ^5^ Also, compounds having a log P less than 1 would struggle to pass from the vehicle to the stratum corneum (SC). ^6^ Solid in oil nanodispersion (SON) is solid drug molecules coated with molecules of lipophilic surfactant (drug-surfactant complex) dispersed as a nano-order particle in the oil phase making the drug permeable into the skin without any physical enhancers or pre-treatments. ^7^ The conventional VEM-SON formula prepared in our lab ^8^ still had low drug aqueous solubility thus less retention time of the drug in aqueous skin tissues that may affect its effectiveness. Many technologies can be adapted including lipid-complex with the drug and assuming optimum HLB via enhancing drug solubility and skin permeation. According to our knowledge; this is the first time to prepare lipid complex for VEM to be further coated in SON taking advantage of lipid biochemistry’s metabolic pathways for targeting. ^9^^,^^10^ Choosing the most suitable lipid as well as predicting the chances of such complex was achieved through the computational prediction (intermolecular Hydrogen bond prediction) using the Cambridge Structural Database’s desktop search interface (CSD) mainly ConQuest which can search all textual, quantitative, and structural data stored in the CSD, as well as Mercury to analyze and draw the results. ^11^

Such novel drug-lipid complex in its SON may have higher anticancer activity, higher skin permeation and deposition with longer retention time in skin epidermis leading to effective topical formulation, less dose frequency, no systemic side effects, and improves patient compliance in treating skin melanoma in early stages.

## Methods

### Predicted Intermolecular Hydrogen Bond search with data analysis

To predict intermolecular hydrogen bonding between VEM and phosphatidylethanolamine (PE), launch conquest (Version 2021.2.0) and open the sketcher by clicking the draw button then start by drawing the functional groups (donor and acceptor according to ChemDraw) for both VEM and phosphatidylethanolamine (PE) ( Table 1). Make connect and define the distance between function groups as well as bond angle, after the search will begin by providing a hitlist that is analyzed using Mercury (Version 2021.2.0). Finally, data are plotted as well as analyzed statistically (descriptive statistics). ^12^ Also, this method is applied to predict the possibility of intermolecular hydrogen bonding between VEM and surfactant (L195) (constituents of the conventional SON) as well as phosphatidylethanolamine (PE) and L195 to calculate the possible number of predicted hydrogen bonds (computational prediction).

**Table 1.** VEM and phosphatidylethanolamine (PE) functional groups added as queries in ConQuest for intermolecular hydrogen bonding probabilities prediction.

VEM functional groups	PE functional groups
N13	NH18
N13	OH19
NH10	N18
NH10	O (1,11)
NH10	O 15
N10	NH18
O (16, 17)	NH18
O (16, 17)	OH19
N11	NH18
N11	OH19
NH11	O 15
NH11	O (1,11)
O18	OH19
O18	NH18
F (25, 27)	NH18
F (25, 27)	OH19
Cl 31	OH19
Cl 31	NH18

### Preparation of VEM-Phosphatidylethanolamine lipid complex (DLC)

Based on the computational structural prediction for the drug and lipid (PE) complex possibility, DLC (Drug-lipid complex) was prepared using the method of solvent evaporation, where an equimolar 1:1 amount of VEM and PE were added to a round bottom flask and dissolved in a mixture of DMSO: chloroform (3:6) then the mixture was stirred using a magnetic stirrer for 3 h at 65 °C. The choice of DMSO: chloroform (3:6) as a solvent was based upon studying the solubility of VEM and PE in different organic solvents like THF (tetrahydrofuran): chloroform, DCM (dichloromethane): chloroform as well as DMSO: chloroform, in different ratios for each. The best solvent in which the drug and the lipid were completely soluble (DMSO: chloroform, 3:6) was chosen. The same way of solvent choice was applied with Rutin: egg phosphatidylcholine complex using DMSO: butanol. ^13^

The solvent was evaporated at room temperature and a desiccator was used to store the resulted lipid film. ^14^ For further confirmation of the best possible stoichiometric ratio for the drug-lipid complex; continuous variation approach (Job’s method) was used to determine the stoichiometric ratio for DLC by preparing DLC with 1:2 and 2:1 drug: lipid complex in addition to the 1:1 ratio mentioned above. The dried lipid films for each molecular ratio (1:1, 1:2, and 2:1) of DLC were redissolved in its solvent’s combination (DMSO: CHCl3, 3:6 ratio) and the pure VEM was dissolved in the same solvents without the lipid complex to determine the absorbance difference between VEM alone and the DLC. Using a UV-visible spectrophotometer, the absorbance of VEM in the DLC was determined at the estimated λ max and subtracted from the total VEM absorbance. The greater the absorbance difference, the better the DLC ratio. ^15^

### Characterization of the prepared VEM-PE complex (DLC)

***FT-IR, Raman spectroscopy, DSC, TGA, P-XRD, and FESEM***

The infrared scans of the samples; pure VEM, PE (phospholipid), physical mixture of VEM and PE as well as the DLC were used to investigate any molecular interactions between the formulation components. Each sample (2 mg) was uniformly mixed with potassium bromide (200 mg) and compressed to make round transparent discs and scanned from 400-4000 cm ^-1^. ^16^

The samples’ Raman spectra for the same samples were acquired using a Senterra Raman Microscope with a wavenumber range of (0-3200) cm ^-1^ and a Raman intensity range of (0-4800) a.u. ^17^

DSC and TGA tests were performed for the same samples using PerkinElmer Thermal Analysis. Each sample was placed on aluminum pans, and heated at a rate of 40 °C/min under an argon atmosphere was used from 40 to 600 °C. ^18^

Using Cu (1.54060 A) as a source of radiation, the P-XRD spectra for the same samples were recorded on a Shimadzu XRD 6000 diffractometer. The data was taken at a step size of 0.02 degrees and a time per step of 0.15 seconds throughout an angular range of 5 to 80 degrees. ^19^

FESEM (CARL ZEISS EVO MA10, Cambridge, UK) was utilized to determine the particle size, surface morphology, and elemental composition of the same samples using gold-coated samples. ^17^

### Preparation of SON loaded with the prepared VEM-PE complex (DLC-SON)

DLC-SON was prepared using the same method applied for the conventional SON prepared previously in our lab ^8^ using the optimum ratio of VEM: L195 (1:6.6). The prepared DLC was dispersed alone in IPM to be used as DLC-control for comparison. In addition to VEM-control previously prepared by dispersing pure VEM powder directly in IPM.

### Characterization of the prepared DLC-SON

Particle size, viscosity measurement, saturated solubility and partition coefficient were estimated for the prepared DLC-SON in comparison to the conventionally prepared VEM-SON as well as the pure VEM and pure DLC applying the same method reported in previous works. ^20^ The in vitro drug release of VEM from its prepared DLC-SON was determined using a vertical Franz cell diffusional system (TP-6 Intelligent Transdermal diffuser; Xi’an Yima Optocelec Co., Ltd) with a receptor part (volume of 15 ml) and a donor part (volume of 3 ml), separated by dialysis membrane (M.W 3500 Da, USA), with a diffusional area of (1 cm ^2^) where fifteen milliliters of PBS containing 1% SDS was used as the medium in the receptor compartment and 300 μL of each of the prepared DLC-SON formula and DLC-control containing 3 mg VEM were separately placed in the donor compartment of the Franz cell instrument. ^21^ As well as the ex vivo skin permeation and deposition studies were determined using the same Franz cells apparatus using the abdominal skin of adult Wister Albino male rats. ^22^^,^^23^

Cell-line study using American Type Culture Collection ATCC provided human Homo sapiens SK-MEL-28 cell lines (stored under liquid nitrogen) (Middlesex, UK). ^24^^,^^25^ The effects of the conventional VEM-SON formula (F3) and the prepared DLC-SON formula on skin melanoma cell viability in comparison to pure VEM were assessed using the MTT assay using different concentrations of each sample (0.025, 0.05, 0.1, 0.35, 0.75, 1.5, 3.12, 6.25, 12.5, 25, 50, and 2.5 μM) at different times (24, 48, and 72 h). ^26^ The percentage of cell inhibition was calculated to estimate half-maximal inhibitory concentration (IC50) for DLC-SON on skin melanoma cell viability in comparison to pure VEM and our conventional VEM-SON formula.

### *In vivo* skin penetration study

***Study design and dose calculation***

The study’s protocol was approved by the Iraqi Center for Cancer Research/Mustansiriyah University’s animal ethical council (approval no. ICCMGR2020-016). The topical dose of rats was determined using the calculated human equivalent dose (HED) of them as described in equation 1 ^27^:

$$\mathrm{HED}\left(\mathrm{mg}\right)=\text{Animal dose}\;\left(\mathrm{mg}\right)\times \left(\frac{\text{Animal}\;\mathrm{Km}}{\text{Human}\;\mathrm{Km}}\right)$$ (1)

Where; Km = species factor (body weight in kg divided by BSA in m ^2^).

The calculated single topical dose of VEM for an adult human is (1.4 mg/kg), while the Km values for an adult human and a rat were 37 and 6, respectively. ^28^ Therefore, by applying equation 1, the VEM dose for rats was 2 mg. A conventional patch (1 cm ^2^) was used to incorporate a certain amount (equivalent to 2 mg) of VEM-SON (F3) and DLC-SON each one separately as well as VEM-control (for comparison). Twelve rats weighing 200-250 grams were anesthetized and divided into three groups (as presented in Table 2).

**Table 2.** Rat groups used for in vivo skin penetration study.

Group no	Trans (dermal) patches	No of rats
Group 1	Trans (dermal) patch containing VEM-control	4
Group 2	Trans (dermal) patch containing VEM-SON	4
Group 3	Trans (dermal) patch containing DLC-SON	4

For each rat in each group *,* the transdermal patch was affixed with a sticker to the skin of the rat’s abdomen after it had been shaved. The rats were then placed in Kent Scientific Corp to keep the patch away from detachment by the rat. From each group; a rat was taken after 1, 4, 8, and 24 h of application of the transdermal patch, and the skin sample was cut, cleaned, and investigated using confocal laser scanning microscopy (CLSM).

***Confocal laser scanning microscopical (CLSM) examination for the treated skin rats***

CLSM (Carl Zeiss LSM700 Microscopy, Germany) was utilized to investigate the time dependent permeability behavior for VEM-SON formula (F3) and DLC-SON in rats’ skin (n=4 per group) and compared with VEM-control (pure VEM dispersed in IPM) after application of fluorescent dye Lucifer Yellow (LYCH) and Fluorescent Isothiocyanate (FITC). The intensity of hydrophilic and lipophilic indicators in the skin has been seen using confocal laser scanning microscopy (CLSM). The main benefit of CLSM is that it allows to see the distribution of the model fluorescent component in the sample without the need for radiolabeling. ^29^ Two types of dyes were used in this study; LYCH (yellow dye) for DLC-SON, and FITC (green dye) for VEM-SON formula (F3), and VEM-control (pure VEM dispersed in IPM) each one separately. Both dye’s permeation through the skin was followed by CLSM at its suitable excitation and emission spectrum. ^30^

The abdomen skin tissue of each rat treated with DLC-SON was mixed with 1% LYCH (yellow dye) for 12 h and then 4% glutaraldehyde in 50 mM phosphate buffer overnight. Then each sample was washed with distilled water. Then, the skin tissue was cut into sections using (a cryostat microtome) with a thickness of around 500μm and investigated with CLSM (LSM700). A diode laser was used to excite the objectives at 405 nm. The emission spectrum (520-569 nm) with a maximum of 548 nm ^30^ (Yellow channel). The same procedure was applied for abdomen skin rat treated with VEM-SON formula (F3) and for VEM-control (each one separately) using FITC dye (green dye). Objectives were excited at 450 nm. The emission spectrum (500-550 nm) with a maximum of 526 nm ^30^ (Green channel). To circumvent the effects of ambient light, all measures were performed in complete darkness. ^31^ Image J software was used to plot luminescence (gray) versus skin diameter (in μm).

## Results and discussion

### Predicted Intermolecular Hydrogen Bond search with data analysis

**Figure 1.**
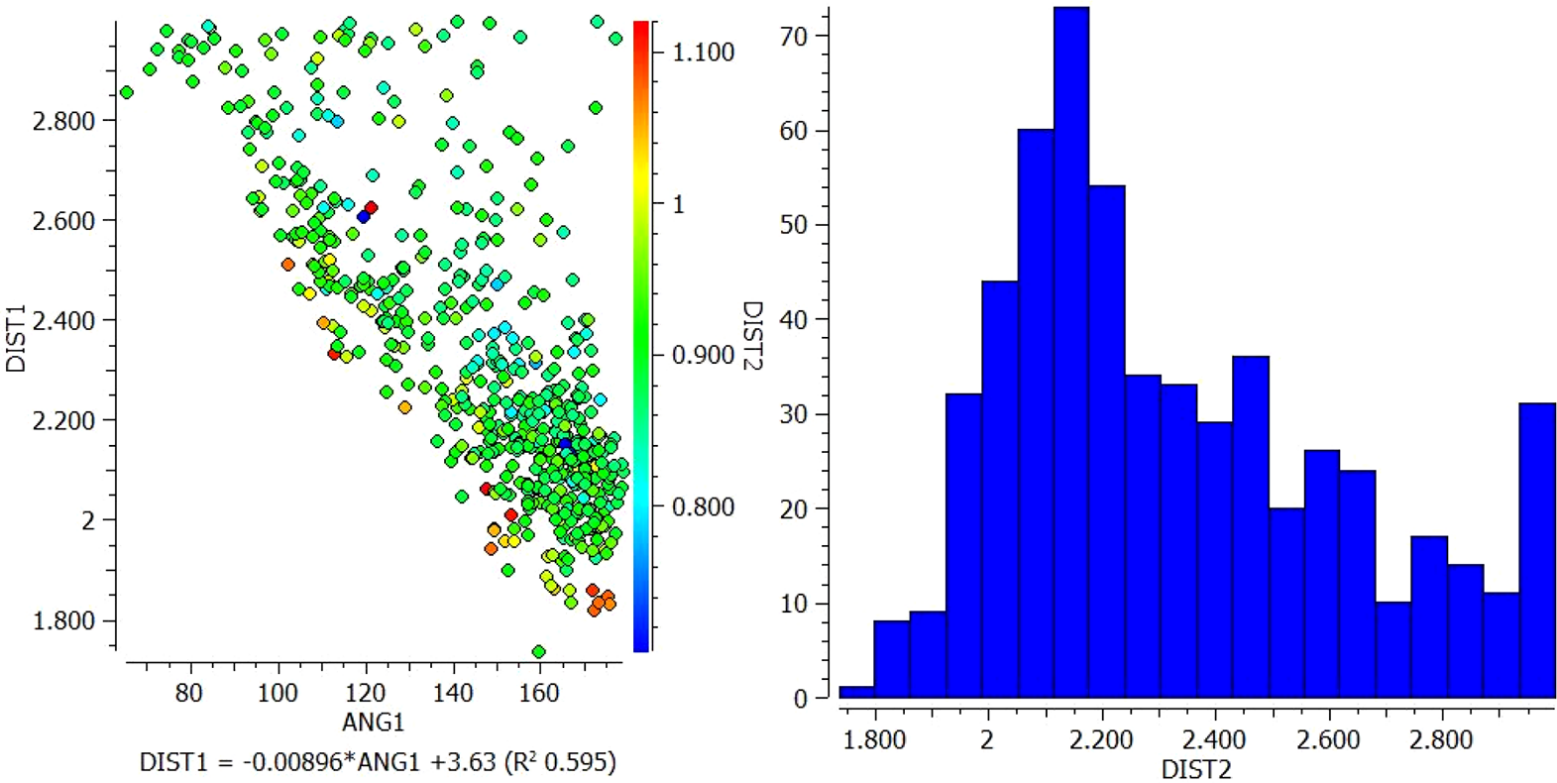
Scatterplot of DIST 1 versus ANG and histogram for N13 of VEM and NH18 of PE.

**Figure 2.**
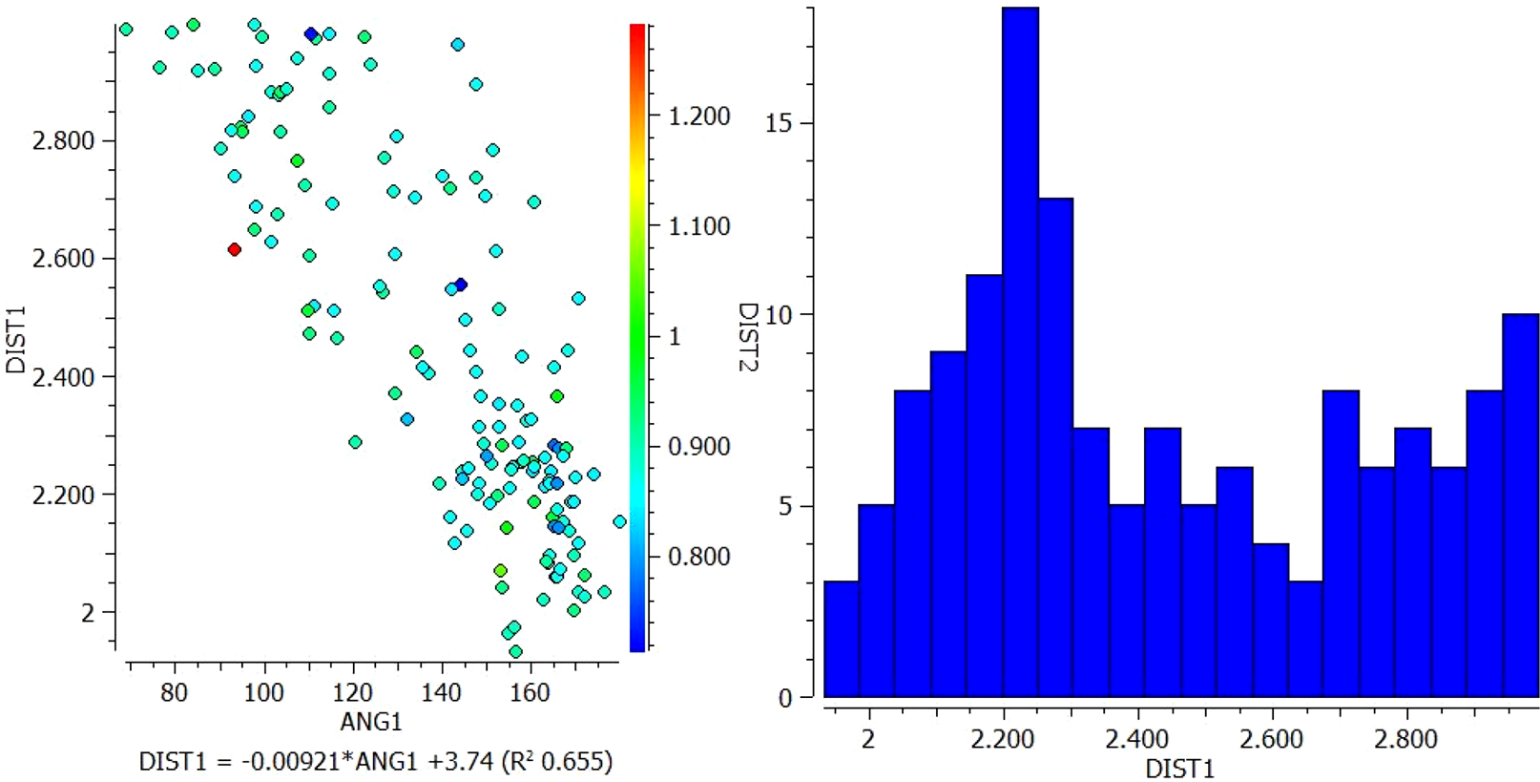
Scatterplot of DIST 1 versus ANG and histogram for O (16, 17) of VEM and NH18 of PE.

**Figure 3.**
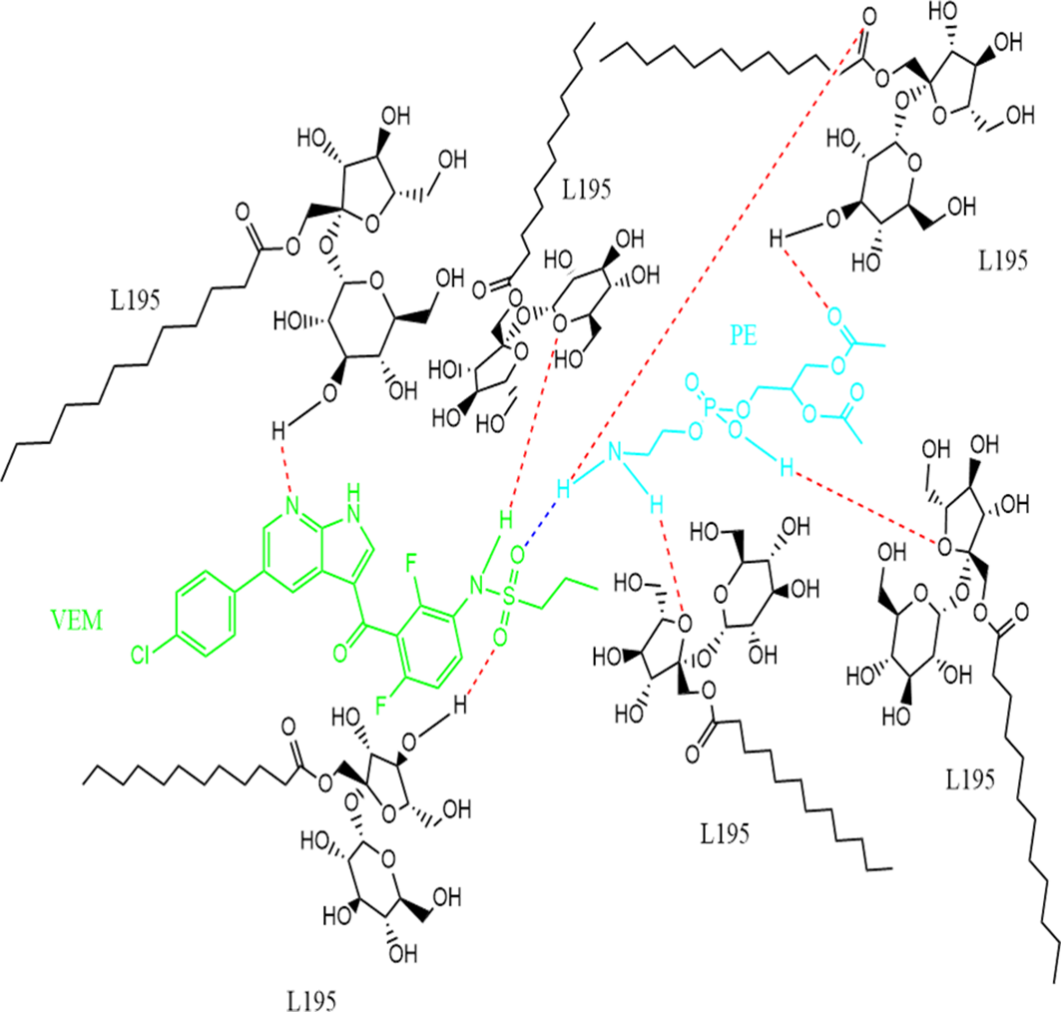
Predicted structure of VEM-PE complex (1:1 ratio) complexed with L195 (1:6.6 ratio) based on CCDC program, where single blue dashed line indicated a strong hydrogen bond between VEM and PE, while the red dashed line indicated strong hydrogen bonds between VEM: PE and L195.

The prediction analysis of hydrogen bond probability was done for the following molecules:

A. Between VEM and PE: Conquest Version 2021.2.0 was used to draw functional groups (donor and acceptor) and then identify the connection to determine intermolecular hydrogen bond distance (DIST) between 1 and 3 A° as well as angle (ANG1) identification ( Table 3), where 12 hydrogen bonds between VEM and PE were predicted which are: Five strong, largely covalent hydrogen bonds were predicted where donor-acceptor distances of 2.2-2.5 Å (they are highlighted in green color). Four moderate, mostly electrostatic hydrogen bonds were predicted where donor-acceptor distances of 2.5-3.2 Å ^32^ (they are highlighted in yellow color). Three anomalously short hydrogen bonds were predicted where Hydrogen bond lengths <2.3 Å ^33^ (they are highlighted in blue color as shown in Table 3).
  Most predicted hydrogen bonds have an angle greater than 130° which is classified as strong while few hydrogen bonds show an angle <130° which are unlikely to be true stabilizing interactions and are unlikely to be maintained in vivo. ^34^ To look for interplay between the strength of intermolecular Hydrogen bonding interaction and hydrogen donor distance (N-H and O-H), the latter was added as a secondary distance (DIS2) value as shown in Table 3 and drawn as a scatterplot ( Figures 1 and 2).
  1. In the interaction between N13 of VEM and NH18 of PE, most of the longer N-H distances (warm colors) correlate with shorter pyridine N but correlate with larger N-H---N angles. However, the shortest N-H distances (blue color) fall approximately in the middle of the range of pyridine N distances and correlate with N-H---N angles of approximately 120° and 165° ( Figure 1).
  2. In the interaction between O (16, 17) of VEM and NH18 of PE, the longer the N-H distance (warm colors) correlates with the long S=O distance and correlates with the shorter N-H---O angle (93°). However, the shortest N-H distances (blue color) fall in the middle of the longer S=O distance and correlate with the N-H---O angle of 144° ( Figure 2).
B. Between VEM and L195: The same method used between VEM and PE was applied for hydrogen bond prediction between VEM and L195. Data (hists or model structures) were analyzed graphically and statistically using CSD Mercury version 2021.2.0, where 15 predicted hydrogen bonds between VEM and L195 were predicted.
C. Between L195 and PE: The same method used between (VEM and PE as well as between VEM and L195) was applied for hydrogen bond prediction between L195 and PE. Data (hists or model structures) were analyzed graphically and statistically using CSD Mercury version 2021.2.0, where 12 predicted hydrogen bonds between L195 and PE were predicted.
  Finally, one molecule each of the drug (VEM) and the lipid (PE) are bound by a single hydrogen bond either by (A) strong hydrogen bonding between N13 of VEM and NH18 of PE, (B) strong hydrogen bonding between O (16, 17) VEM and NH18 of PE, (C) strong hydrogen bonding between NH11 of VEM and O (1,11) of PE, or by (D) strong hydrogen bonding between O18 of VEM and NH18 of PE. This, explains the stoichiometric ratio of VEM: PE in 1:1 ratio. While the binding of the surfactant to VEM: PE occurs through many hydrogen bonds. Hence, this indicates the ratio of VEM: L195 of the conventional VEM-SON formula F3 (1:6.6). Figure 3 shows the predicted structure of DLC-SON.

**Table 3.** Predicted intermolecular hydrogen bond in CSD model structures and realized hydrogen bonds distances and angles for VEM and PE functional groups (models mean±SD).

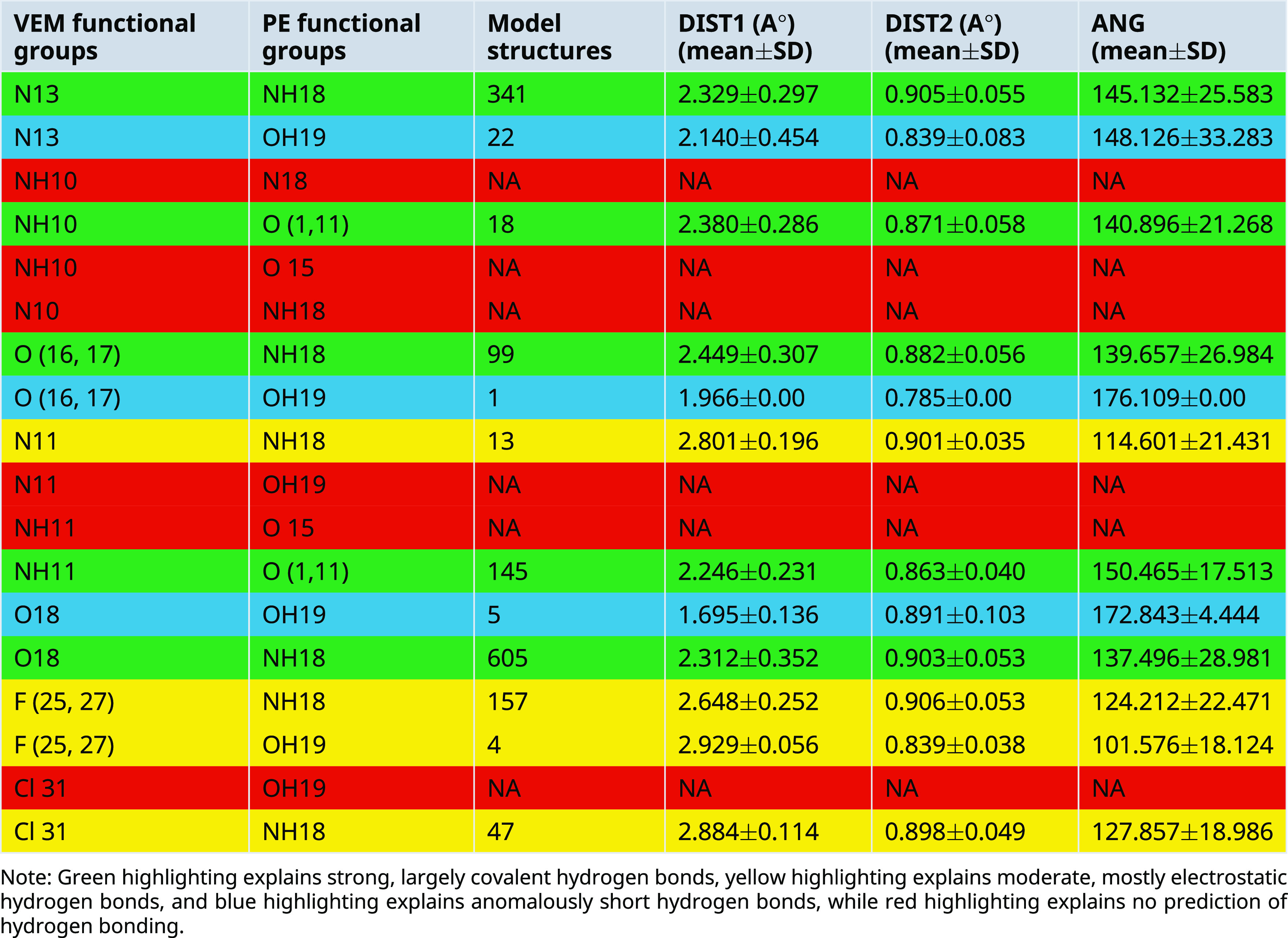

### Preparation of VEM-Phosphatidylethanolamine lipid complex (DLC)

**Figure 4.**
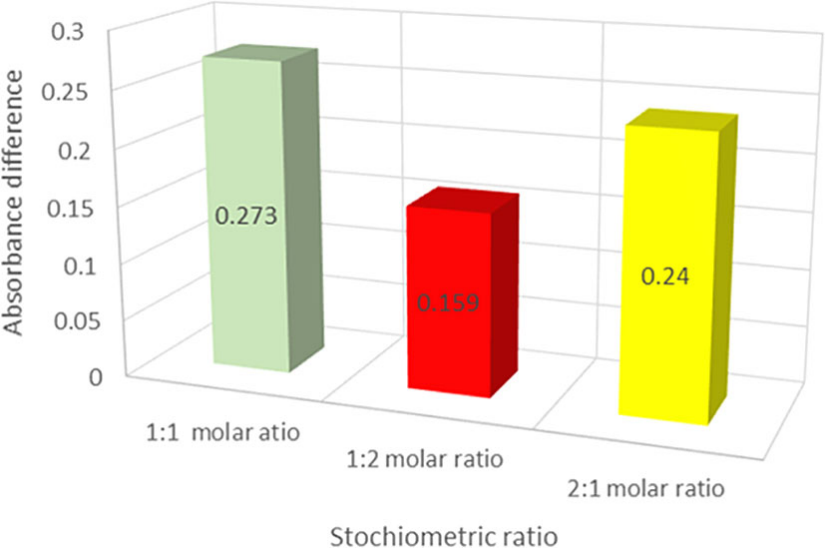
The absorbance differences of the various drug: lipid ratios of the DLC, (values are mean ±SD) (n=3).

The DLC was generated based on the computational structural prediction for the drug and lipid complex possibility using the solvent evaporation method. The influence of the drug: lipid ratio on the formation of the DLC was investigated using the continuous variation approach. The UV absorbance of the DLC was measured at a different drug: lipid molar ratios (1:1, 1:2, and 2:1). The relationship between the drug: lipid molar ratios and the net difference in absorbance (∆A) is shown in Figure 4. The absorbance difference (∆A) between total VEM added and VEM in the lipid complex was found to be larger at the drug: lipid mole ratio of 1:1 than at other ratios (1:2, and 2:1), indicating that more VEM was conjugated with PE at the 1:1 ratio, thus proves the results of computational prediction of hydrogen bonding between VEM and PE, comparable to probucol results. ^35^ As a result, the drug: lipid (1:1) ratio of the DLC was chosen for further investigation.

### Characterization of the prepared VEM-PE complex (DLC)

**Figure 5.**
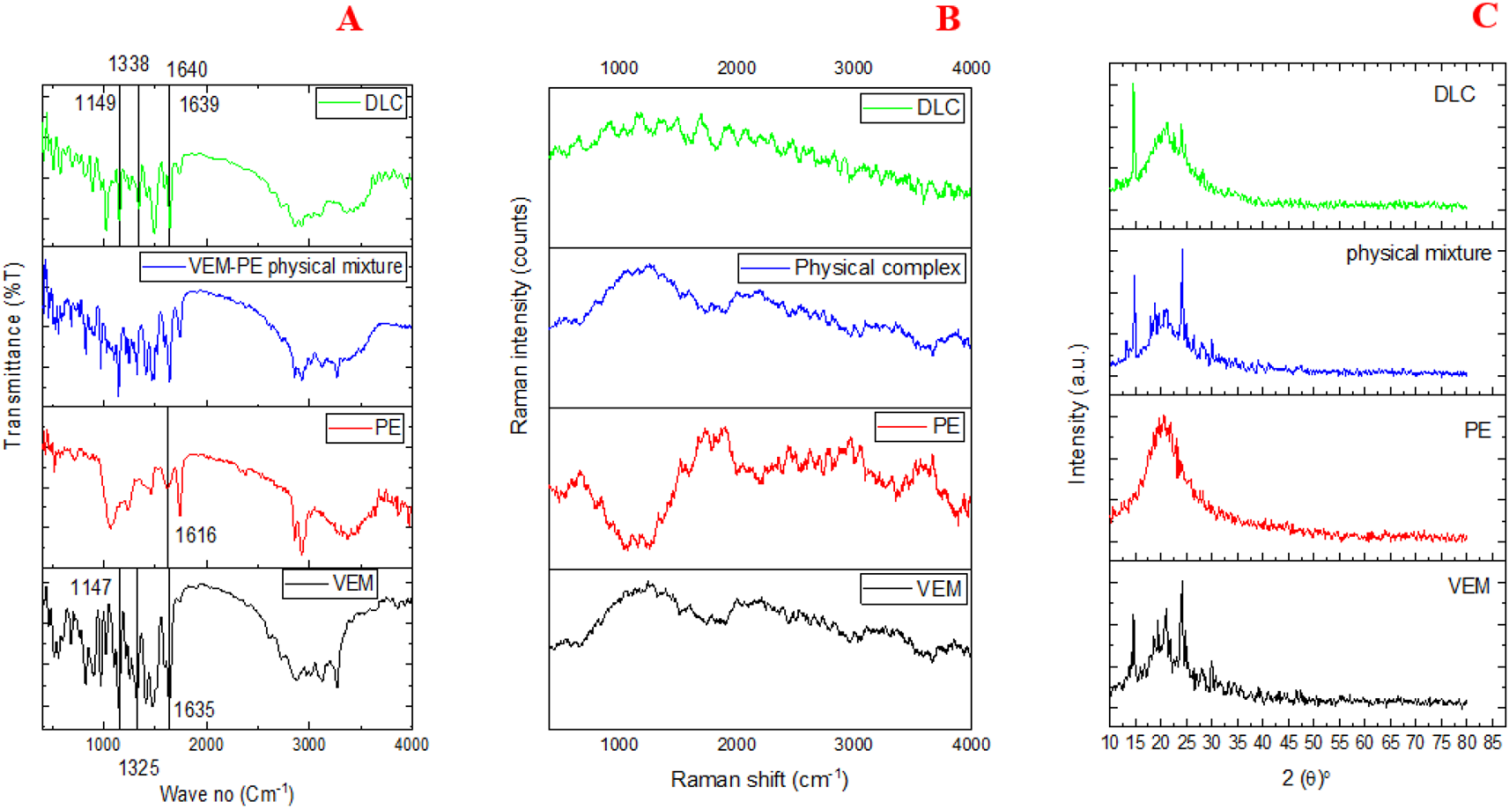
Characterization of VEM-PE complex (DLC)(A) FT-IR spectrum, (B) Raman spectroscopy, and (C) PXRD of VEM, PE, physical mixture of VEM and PE, and DLC.

In Figure 5A, the IR spectrum of VEM exhibited distinct absorption bands (ν, cm ^-1^): 1734 (C=O), 1325,1147 (SO2), and 1635 (C=N) agreed with the reported data. ^36^ While the PE spectrum exhibited a distinct absorption band (ν, cm ^-1^): 1616 (N-H) similar to the reported. ^37^ DLC spectrum shows the change of the following VEM characteristic absorption bands (ν, cm ^-1^): 1639 (C=N) (4° shifting), 1338, 1149 (SO2) (13°, 2° shifting) as well as shows the change of the PE characteristic absorption bands (ν, cm ^-1^): 1640 (N-H) (26° shifting). The IR spectra of DLC corroborate CCDC’s H-bond prediction (Conquest and Mercury) since these functional groups showed strong hydrogen bonding between N13 of VEM and NH18 of PE, and strong hydrogen bonding between O (16, 17) VEM and NH18 of PE.

The differences in the vibrational energy of crystalline and amorphous materials can be detected by IR and Raman spectroscopy. Sharp peaks are produced by crystalline materials, while broader peaks are produced by amorphous materials. ^38^ In Figure 5B, VEM’s Raman spectrum exhibited sharp peaks indicative of its crystalline nature, whereas DLC’s Raman spectrum exhibited broader peaks indicative of its amorphous nature. The Raman spectra of paracetamol, flufenamic acid, and imipramine hydrochloride all yielded similar results. ^39^

Figure 5C displays the powder X-ray diffraction patterns of VEM, PE, a physical combination of VEM and PE, and DLC. Due to its amorphous nature, PE displayed a broad peak, whereas VEM showed intense diffraction peaks, implying that the drug material is crystalline. ^40^ The physical mixture of VEM and PE exhibited the same diffraction peaks as VEM but with a lower intensity, indicating the physical mixture’s semicrystalline nature; this difference can be attributed to the grinding effect during the physical mixture’s preparation. On the other hand, DLC revealed that the crystalline peaks of VEM disappeared, implying that the substance had completely amorphized may be due to complex (H-bonds formation). ^40^

**Figure 6.**
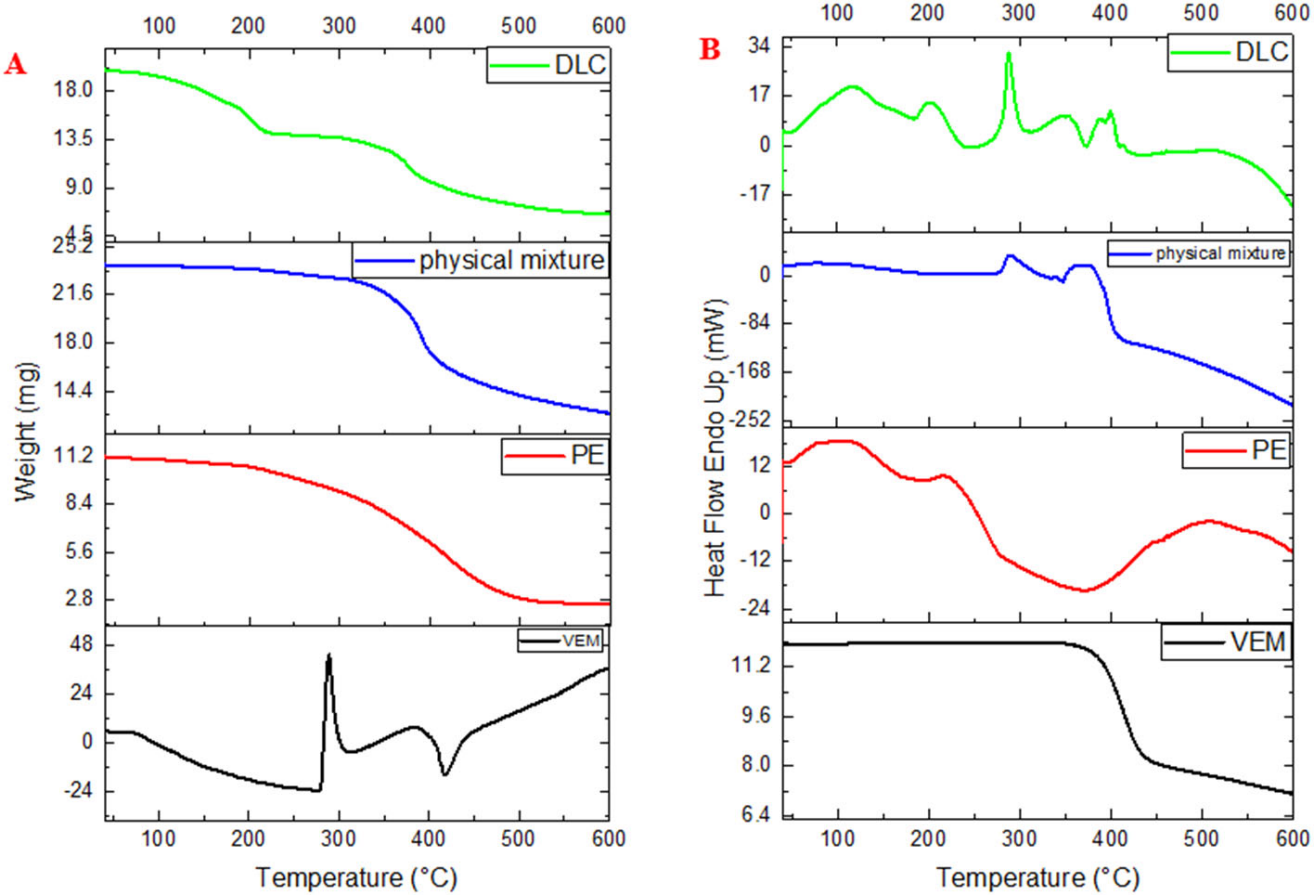
(A) DSC and (B) TGA of Pure VEM, Pure lipid (PE), physical mixture of VEM and PE, and DLC (drug-lipid complex).

Figures 6 (A) illustrate the DSC thermograms of VEM, PE, physical mixture of VEM and PE, and DLC respectively. Pure VEM exhibited sharp melting endotherms at approximately 272 °C, comparable to the melting point of VEM crystals, ^3^ the PE, on the other hand, did not exhibit any sharp melting event, supposedly due to its amorphous nature. The glass transition temperature (Tg) is thought to be the small endotherm peak at 196 °C of PE. ^41^ Additionally, the DSC curve of DLC revealed that the original VEM peaks had vanished, implying that the interaction (complex) between VEM and PE occurred primarily through hydrogen bonds formation. ^42^ The physical mixture, on the other hand, showed the same Tg peak of PE, which could be due to partial interaction between VEM and PE in the presence of rising temperature during sample analysis, as seen with mefenamic acid. ^14^

Figures 6 (B) illustrate the TGA thermograms of VEM, PE, physical mixture of VEM and PE, and DLC respectively. The TGA curve of VEM demonstrates that the drug begins to decompose at 350 °C (single-stage decomposition/single weight loss region). The TGA curve for PE revealed three distinct stages of decomposition/three distinct regions of weight loss. The TGA curve of the physical mixture revealed two stages of decomposition/two regions of weight loss. DLC’s TGA curves showed multiple stages of decomposition/multi-weight loss regions (five regions), in contrast to VEM’s single mass loss region. Each transition point signaled the ignition of a new component. The TGA weight loss % (using 12 mg sample) each of VEM, PE, physical mixture, and DLC was begun at 100%, 95%, 98%, and 95% correspondingly at 350°C, 100°C, 280°C, and 100°C respectively. Thus, TGA was a good way to figure out how much weight VEM and DLC lost. ^43^ According to the calculated value of the ignition index (Di) ^44^; the higher Di value means a more efficient and stable decomposition process where, DLC had a higher value (5.3) in comparison to PE, VEM, and physical mixture (4.7, 0.71, and 0.58 respectively).

**Figure 7.**
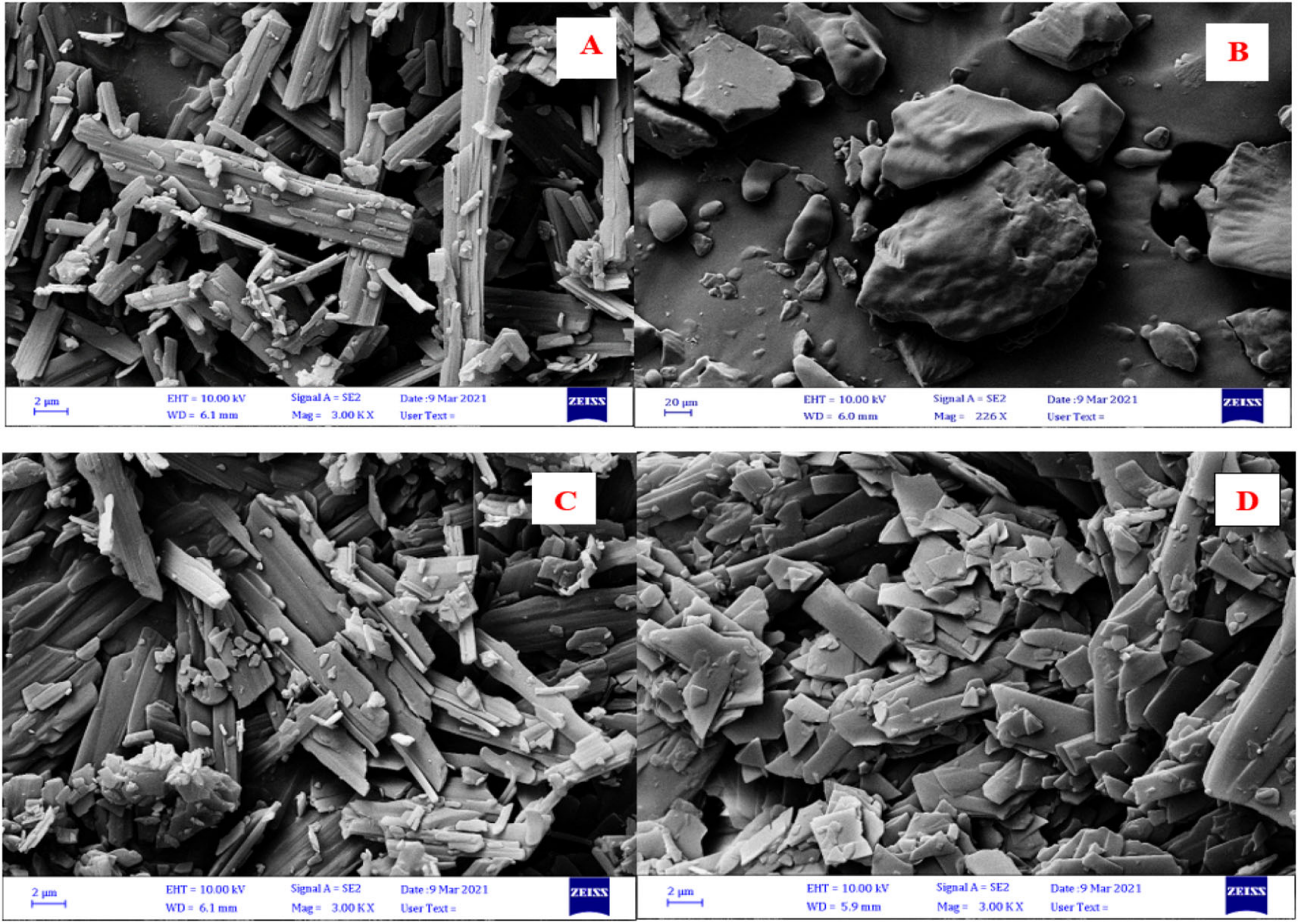
FESEM images of (A) VEM, (B) PE, (C) physical mixture of VEM and PE, and (D) DLC.

The surface morphology of VEM, PE, a physical mixture of VEM and PE, and DLC are shown in Figure 7 (A, B, C, and D). VEM’s surface morphology revealed rod-shaped crystals, whereas DLC revealed a complete lack of crystalline nature of VEM. This is due to the drug’s crystallinity being completely concealed as a result of complexation with PE, which creates polymorphic shifts in the drug’s crystal habit, turning it amorphous. ^40^ Additionally, the FESEM imaging results were found to be inconsistent with the FT-IR, Raman spectroscopy, DSC, TGA, and PXRD results for the purpose.

### Characterization of the prepared DLC-SON

The prepared drug-lipid complex (DLC) was utilized to prepare SON formula named as DLC-SON and characterized. It showed a particle size of 556.9 nm compared to DLC-control and VEM-SON (3.995×10 ^4^ and 484.5 nm respectively). DLC-SON showed a larger particle size due to the complexation (between VEM and PE) in DLC, which further leads to larger molecular weight molecules. While DLC-SON showed a smaller particle size in comparison to DLC-control (P<0.05) since L195 is highly soluble in the organic phase (cyclohexane) and has a low HLB value (≈ 1). ^45^ The same results were observed with Boswellic acid conjugated vesicles. ^46^

VEM was classified as a Class IV drug (low solubility and low permeability). ^47^ The aqueous solubility of VEM in its DLC was significantly (P˂0.05) higher than pure VEM powder (64.6 mg/mL versus 0.9 μg/mL). This increase is due to the amorphous characteristics of DLC. The same result was observed with probucol–phospholipid complex. ^35^ The aqueous solubility of VEM in DLC-SON (59.4 mg/ml) is significantly (P<0.05) higher than its aqueous solubility in VEM-SON (3.7 mg/ml) due to the amorphous characteristics of DLC as well as the aqueous solubility of VEM in DLC (64.6 mg/ml) is higher than its aqueous solubility in DLC-SON (59.4 mg/ml) which may be attributed to the masked DLC by complexation with L195 (HLB=1). Moreover, the aqueous solubility of VEM in DLC-control (112.1 mg/ml) is significantly (P<0.05) higher than its aqueous solubility in DLC-SON (59.4 mg/ml) therefore, the dispersion of DLC in IPM (DLC-control) increased drug aqueous solubility by two folds. Similar results observed with IPM-based microemulsion of progesterone and indomethacin due to higher solubility of ester groups of the lipid (PE) in IPM, ^48^ and with diclofenac prodrugs as well. ^49^

Pure VEM, as well as DLC alone, gave a low partition coefficient value (Log P=0.65 and 0.33 respectively). The slightly decreased log P was because DLC showed significantly improved solubility in water, which may be explained by the amorphous character of DLC and the amphiphilic nature of PE. ^50^ The complexation of VEM with PE changes the aqueous solubility and log P of VEM may be attributed to intermolecular hydrogen bond formation between VEM and PE (as predicted by ConQuest and Mercury programs and indicated by DLC characterization). Too many hydrogen bond donors/acceptors, on the other hand, can negatively affect the drug’s membrane partition and permeability. These polar groups can reduce the affinity of the drug for the hydrophobic membrane region while simultaneously increasing the water desolvation penalty during drug penetration. ^51^ The dispersion of VEM and DLC separately in IPM (VEM-control and DLC-control respectively) significantly (P<0.05) increased the partition coefficient (Log P=0.89 and 0.87 respectively) in comparison to pure VEM and pure DLC (Log P=0.65 and 0.33 respectively) because IPM is an aliphatic ester with a molecular weight of 298 g/mol and Log P 2.09 ^52^ but still the Log P in the VEM-control and DLC-control insufficient which makes VEM unable to penetrate the skin (i.e., less than adequate Log P range for skin penetration which was reported at least between (1-3). ^53^ The complexation of VEM with L195 and DLC with L195 which were further dispersed in IPM (VEM-SON and DLC-SON) significantly increased (P<0.05) partition coefficient in comparison to VEM-control and DLC-control (Log P=1.08 for VEM-SON and 1.07 for DLC-SON), thereby both VEM-SON and DLC-SON may pass stratum corneum efficiently. This can be attributed to the low HLB of L195 (≈1) and its long fatty acid chains in the sucrose ester (C12 alkyl chain surfactant). ^54^ Hence, the lipid complexation with VEM improves the aqueous solubility of the drug as well as its skin permeation upon its dispersion as SON formula.

No significant changes in apparent viscosities between the optimum VEM-SON and DLC-SON. Complex viscous behavior is observed, with two different regions. The first one shows viscosity values independent of shear rate, while the second one reveals a shear-thinning behavior that appears at higher shear rates. This would yield a shear-thinning behavior (non-Newtonian behavior) due to the progressive orientation of the micellar aggregates in the flow direction with higher shear rates. ^55^ The complexation of VEM with PE in the prepared DLC-SON had no effect on the viscosity of its SON same as VEM in the optimum VEM-SON.

**Figure 8.**
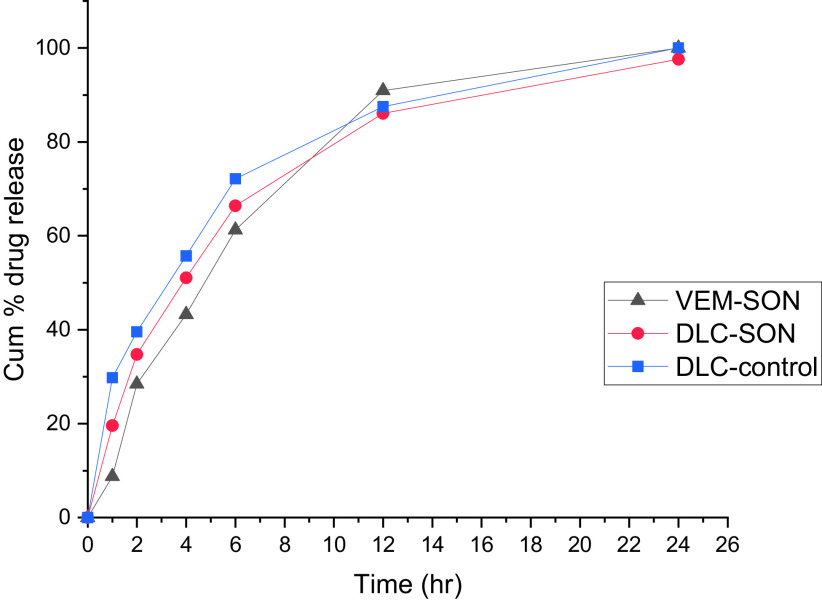
In vitro VEM release from DLC-SON and DLC-control in comparison to VEM-SON in PBS containing 1% SDS.

Figure 8 shows the VEM release from DLC-control and DLC-SON in comparison to the optimum VEM-SON. Pure DLC dispersed in IPM (DLC-control) showed initial release (29.8% within 1 h) compared to DLC-SON and VEM-SON which showed 19.6% and 9% VEM release respectively after 1 h. The initial release in DLC-control is due to its low viscosity while in DLC-SON the initial release may be due to the presence of some DLC particles free or deposited on the outer surface of the surfactant L195 and causing quick efflux of the free drug that considered as the desired effect to ensure the initial therapeutic action of the drug. DLC-SON showed a higher % initial release (P>0.05) compared to VEM-SON (19.6% VS. 9%) due to the amorphous characteristics of DLC that provided better aqueous solubility, which might enhance the in vitro VEM release. While VEM-SON showed a higher % release (P>0.05) after 12 h due to smaller particle size (484.5 nm) compared to DLC-SON (556.9 nm). The increase in particle size led to less surface area exposed to the dissolution medium in addition to the non-significantly lower viscosity of VEM-SON. ^56^ These results were supported using DD solver since DLC-control shows maximum dissolution efficiency (DE) at 12 h and 24 h (62.6 and 78.2 respectively) compared with VEM-SON (54.7 and 75.1 respectively) and DLC-SON (58.1 and 75.6 respectively) (P>0.05). DLC-SON showed the dissimilarity factor (ƒ1) equal to 7.63 and the similarity factor (ƒ2) equal to 63.74 in agreement with FDA guidelines. Similar release patterns were obtained for both DLC-SON and the optimum VEM-SON. The in vitro release kinetic analysis with DDsolver reveals that DLC-SON and DLC-control have the highest correlation coefficient (R ^2^) fit with First-order like the optimum VEM-SON. According to the Korsmeyer-Peppas model, DLC-SON and DLC-control have diffusion exponent (n<0.45) indicating Fickian diffusion case one which means the results of a molecular diffusivity and a concentration gradient determines drug mass transfer flow in this mechanism. ^57^

**Figure 9.**
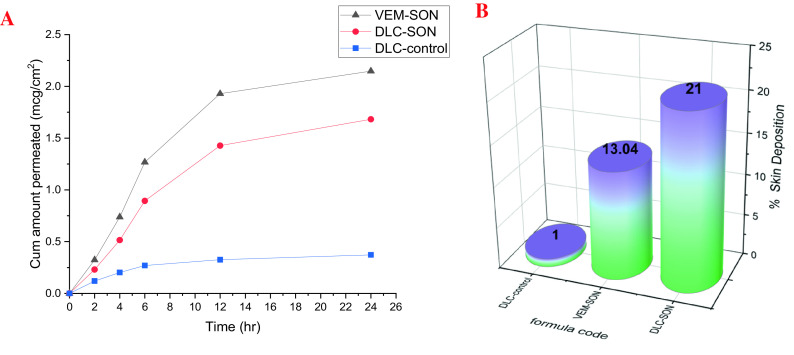
Ex vivo studies of VEM from DLC-SON and DLC-control in comparison to the optimum VEM-SON (A) ex vivo permeation study (B) Skin deposition study.

The ex vivo permeation study by Franz cell diffusional apparatus was utilized to evaluate VEM permeation from DLC-control and DLC-SON in comparison to the optimum VEM-SON utilizing rat abdomen skin as a permeation barrier. The results in Figure 9A show that VEM in DLC-SON has significantly (P<0.05) higher skin permeation than DLC-control, where DLC-control showed only 26.5% drug permeated after 24 h ( Table 4). This could be due to the large particle size in the DLC-control, higher aqueous solubility and lower partition coefficient (Log P) which is inversely proportional to skin permeation. ^58^ VEM-SON showed higher drug permeation than DLC-SON due to a smaller particle size (484.5 nm) compared to DLC-SON (556.9 nm) as well as to its slightly lower viscosity. These results in consistent with the obtained in vitro release data using a dialysis membrane. The values of the permeation parameters such as permeation rate or transdermal VEM flux at steady state (J _ss_, μg/cm ^2^/h), permeability coefficient (KP, cm/h), and enhancement ratio (ER) rose dramatically for DLC-SON in comparison with pure DLC (DLC-control). This emphasis the role of SON technology in improving the permeation of high molecular weight compounds. ^59^ The Jss and time taken for the diffusion flow to become stable (T _lag_) were used as indications for the skin permeation of VEM. The lag time is significantly (P<0.05) shorter for drug from DLC-control (0.1 h) than from DLC-SON (0.96 h) and VEM-SON formula F3 (0.92 h); because of significant (P<0.05) low viscosity in comparison to SON formulations which contain the surfactant L195. ^60^

**Table 4.** Ex vivo permeability parameters of VEM from DLC-SON, the optimum VEM-SON and DLC-control.

Formulas	Jss (μg/cm ^2^/h)	T _lag_ (h)	KP (cm/h) *10 ^-3^	ER	% VEM permeated/24 h
DLC-control	88.93	0.10	8.89	1.0	26.5
DLC-SON	192.52	0.96	19.25	2.16	63.0
VEM-SON	258.38	0.92	25.84	2.65	71.6

Figure 9B depicts the amount of DLC retained in the skin from DLC-SON and DLC-control compared to the optimum VEM-SON after 24 h of permeation experiments. The action of L195 on skin resulted in higher skin deposition of DLC-SON and the optimum VEM-SON compared to DLC-control which gave the lowest deposition. This provided a way for continued drug administration for a longer length of time. ^61^ The permeation within skin (%skin deposition) was low for DLC-control in comparison to SON formulations because of larger particle size of DLC-control (39 μm) that hampered the penetration of DLC-control through the skin hence its lower deposition. On other hand, the presence of phospholipid PE in DLC-SON formula allows for continuous drug administration by interacting between the biodegradable and biocompatible phospholipid PE in DLC-SON and the endogenous phospholipids of the epidermal layer, which permits VEM penetration and fusing into the deeper skin layer and causes its accumulation there.

### Cell-line study

**Figure 10.**
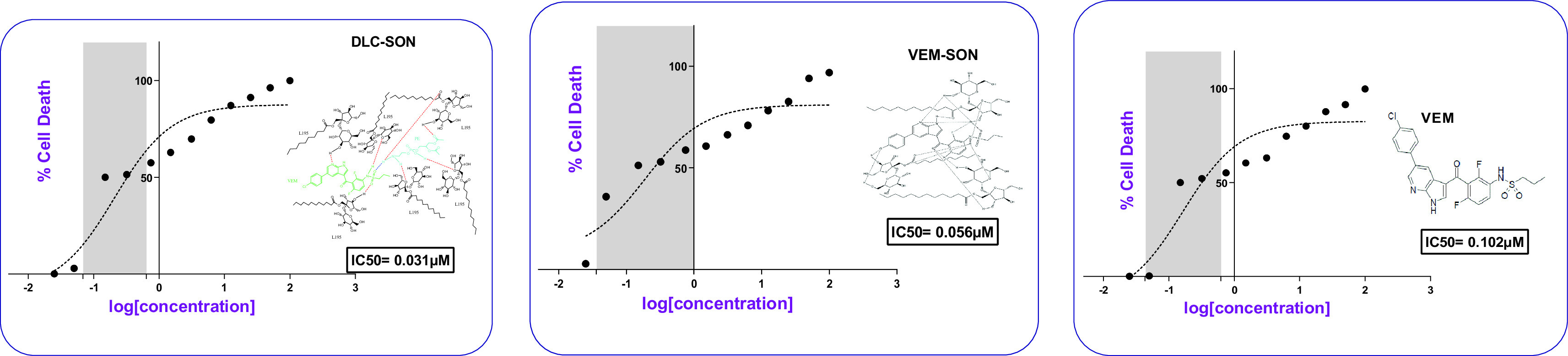
Dose-response curves for IC50 determination for the prepared DLC-SON, VEM-SON, and pure VEM in SK-MEL-28 cells.

The dose-response curve for the prepared conventional VEM-SON, DLC-SON, and pure VEM in melanoma cells (SK-MEL-28) was constructed in this study by plotting the percentage of cell death versus log concentration as shown in ( Figure 10). DLC-SON, VEM-SON, and pure VEM had IC50 values in SK-MEL-28 cells of (0.031, 0.056, and 0.102 μM respectively). The results revealed that the potency of the prepared DLC-SON is significantly higher (P<0.05) than the prepared VEM-SON (by 1.8 folds), and the pure VEM (by 3.3 folds), since a very low concentration can inhibit half of the total number of total viable cancer cells. This indicates the capacity of the prepared DLC-SON in improving the anticancer activity (low IC50) of VEM.

### In vivo skin penetration study

When comparing the results available from various experiments’ skin models, caution should be exercised in interpreting the results. Although guinea pigs, mice, and rats are frequently used as models, their skin is significantly thinner than human skin for comparable evaluation. The crossing step into the SC, the hydrophobic portion of the skin, is generally recognized as the rate-limiting step of penetration into the skin. ^62^

**Figure 11.**
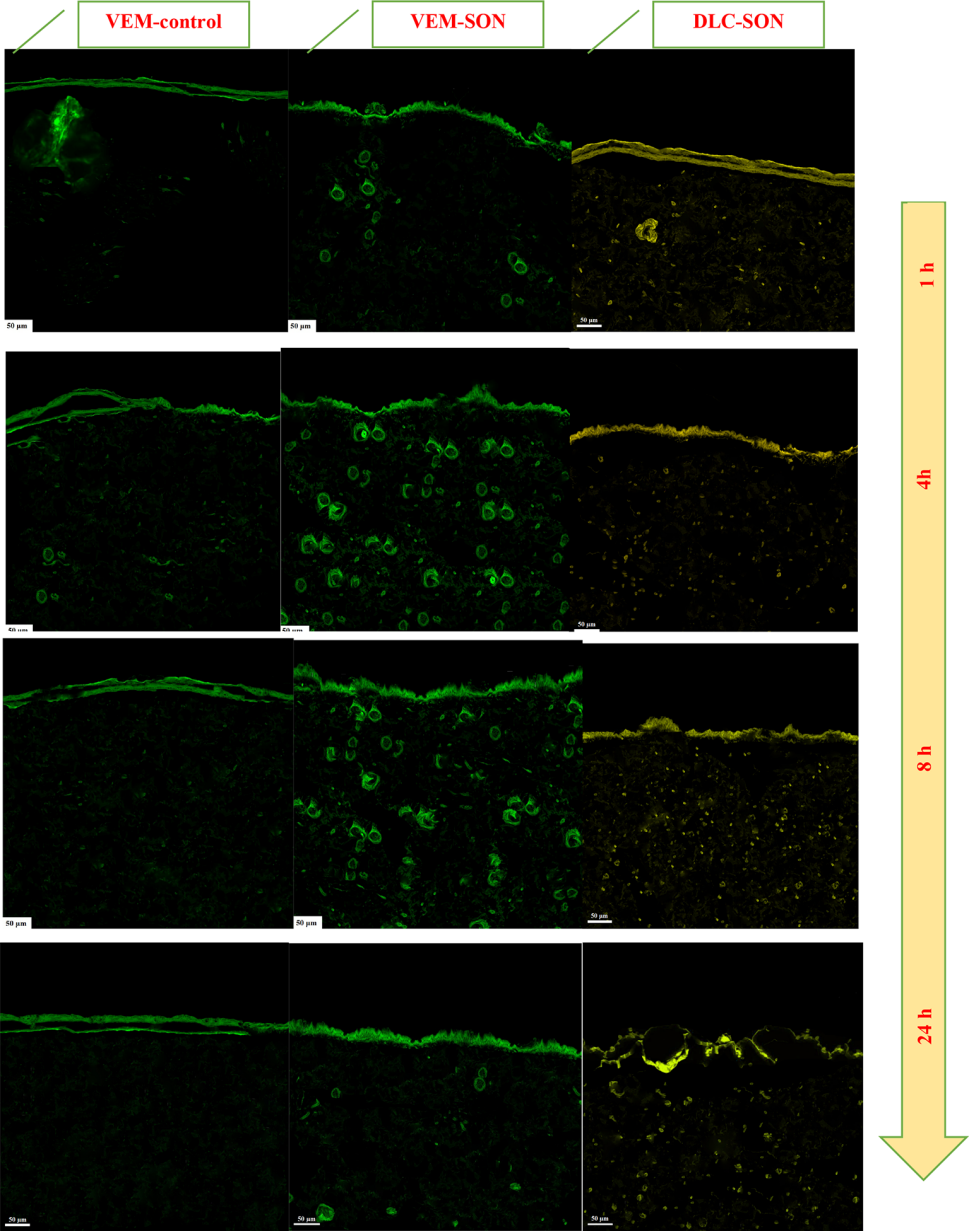
CLSM for the treated rats' skin sections treated with (A) VEM-control, (B) the prepared VEM-SON formula, and (C) DLC-SON, after application of LYCH and FITC (scale bar: 50 μm). Samples were applied at 1, 4, 8, and 24 h.

The CLSM images of the rat skin sections (as a model study) treated with VEM-control, the prepared VEM-SON formula, and DLC-SON, after application of LYCH and FITC dyes are shown in ( Figure 11). In VEM-SON, (where the drug was coated with surfactant L195 and further dispersed in IPM): it is believed that the VEM-L195 complex penetrates the skin concurrently with the IPM permeation. According to reports, IPM’s permeation-enhancing effect is primarily on the SC, increasing the diffusivity within the SC and/or the partition coefficient between the SC and the vehicle for both the drug and the solvent. ^52^ As a result, the hydrophobic SC can be penetrated by VEM using this delivery system. Surfactant molecules would be liberated at the interface between SC and epidermis since the surfactant’s (L195) higher intensity to the oil phase. To demonstrate this point, the skin loaded with VEM-SON and VEM-control were treated with 1% FITC (green dye), whereas the skin loaded with DLC-SON were treated with 1% LYCH (yellow dye).

**Figure 12.**
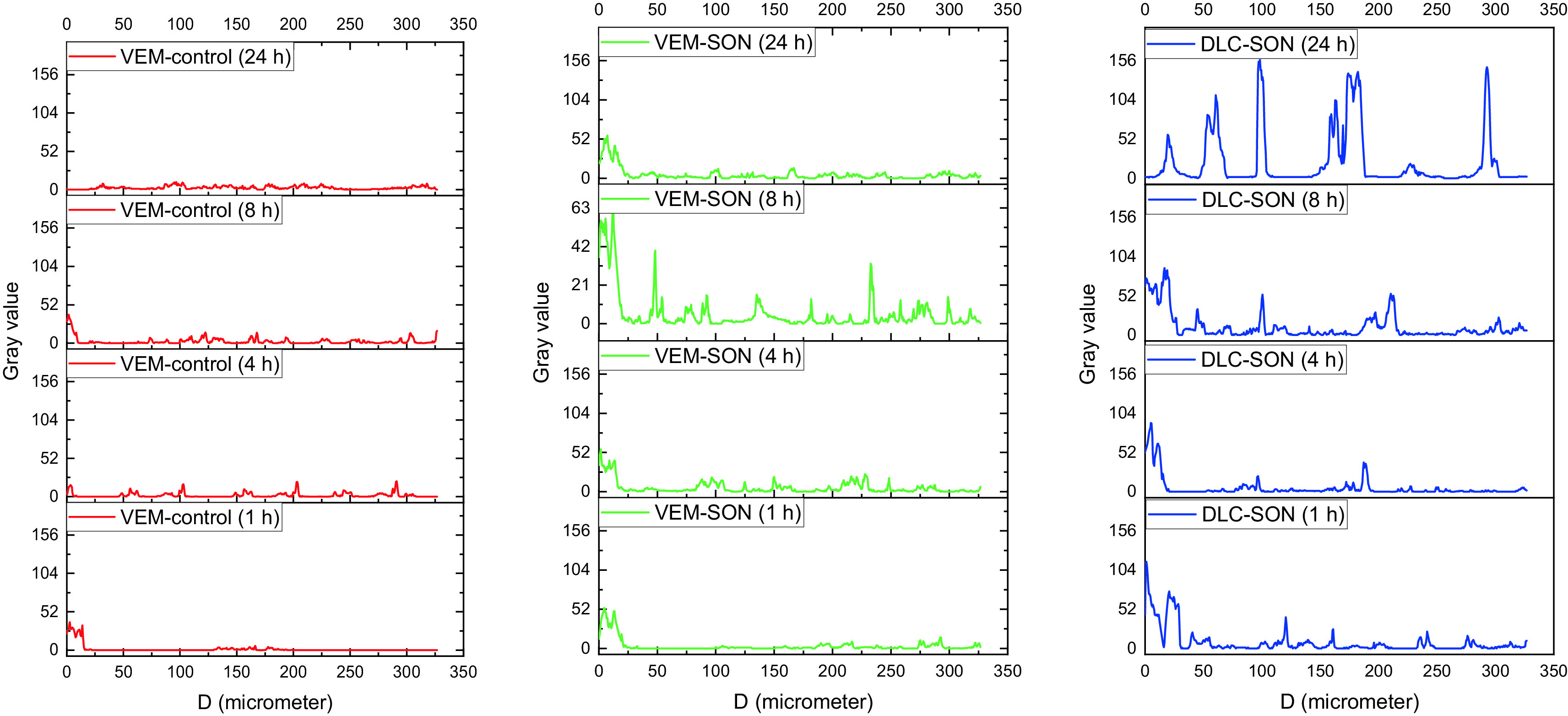
The luminescence or gray value versus skin diameter (in μm) for the treated rats' skin sections treated with VEM-control, the prepared VEM-SON, and DLC-SON, after application of LYCH and FITC in the CSLM images were quantified using the software ImageJ.

The green fluorescence dye derived from the FITC-treated skin sections loaded with VEM-SON was gradually increased at SC (Distance<20 μm) and dermis (Distance>20 μm) with time (1-8 h) but it decreases after 24 h in the dermis (Distance>20 μm) ( Figure 12). These results indicated that VEM-SON seems to readily cross the SC and infiltrate the dermis and epidermis (through intercellular routes as reported ^63^ but it has lower luminescence (a short retention time after 24 h because of the low aqueous solubility of VEM in aqueous skin layers. On other hand, the green fluorescence dye derived from the FITC-treated skin sections loaded with VEM-control was gradually decreased with time at SC and increased gradually in the epidermis with time (1-8 h) and disappeared approximately after 24 h ( Figure 12), It was proposed that IPM enhanced the permeation of VEM-control into the skin, as well as IPM’s cohesive skin-penetration effects. More FITC was delivered into the skin by VEM-SON than VEM-control (P<0.05), a result consistent with insulin in its SON. ^64^ These results are in consistent with our in vitro skin permeation study.

VEM-SON nanoparticles were deposited into the deep skin layers as small “aggregates” and appeared as cubic shape nanoparticles due to their crystalline state. The same result was observed with phospholipid-PEG and cholesterol PEG decorated gold nanorods. ^65^

In DLC-SON, CSLM images ( Figure 11) showed uniform distribution of DLC in the epidermis and dermis where DLC revealed a complete lack of crystalline nature of VEM. This is due to the drug’s crystallinity being completely concealed as a result of complexation with lipid, which creates polymorphic shifts in the drug’s crystal habit, turning it amorphous and agreed with our DSC analysis. The yellow fluorescence dye derived from the LYCH-treated skin sections loaded with DLC-SON was gradually decreased at SC (Distance<20 μm) and increased gradually in the dermis (Distance>20 μm) with time (1-8 h) ( Figure 12). These results also indicated that DLC-SON also readily cross the SC and infiltrate the dermis and epidermis (in intercellular routes ^63^ but it has a longer retention time after 24 h in the dermis (unlike VEM-SON) because of the higher aqueous solubility of DLC in aqueous skin layers. These results were confirmed by strong yellow fluorescent staining of the dermis treated observed after 24 h in comparison to little green fluorescence staining was observed with VEM-SON. These results confirmed that the phospholipid (PE) of DLC is the driving force that made DLC have particularly longer retention duration in skin dermis, through utilizing SON, where a good hydrophilic-lipophilic (HLB) balance can be attained giving the DLC-SON good permeation, longer retention time in skin and higher anticancer activity (lower IC50).

## Conclusion

This study involved (for the first time according to our knowledge) the preparation of VEM-lipid complex using PE as a lipid in 1:1 ratio and formulating it as solid in oil nanodispersion. Such preparation highly improved the anticancer activity (low IC50) of the drug, as well as enhanced skin permeation and deposition with longer retention of the drug in a solubilized form in the skin dermis which makes it a good candidate to treat skin melanoma at the early stage of diagnosis to eliminate the need for surgery and overcome the serious side effects of the high dose oral regimen.

## Data availability

Zenodo: CLSM, ConQuest and Mercury results, https://doi.org/10.5281/zenodo.6639033. ^66^

This project contains the following underlying data:

- CLSM.docx (microscopy images)
- ConQuest and Mercury steps

Zenodo: all underlying data behind tables and graphs presented in Excel sheets, https://doi.org/10.5281/zenodo.6762250. ^67^

This project contains the following underlying data:

- Excel sheets as XY data of all figures

## Reporting guidelines

Zenodo: The arrive guidelines for animal reporting according to the ethical approval, https://doi.org/10.5281/zenodo.6762088. ^68^

Data are available under the terms of the Creative Commons Attribution 4.0 International license (CC-BY 4.0).
