# Supplementary material for: Modified solid in oil nanodispersion containing vemurafenib-lipid complex- in vitro/ in vivo study
Source: F1000Res. 2022 Sep 7;11:841. Originally published 2022 Jul 27. [Version 2] doi: 10.12688/f1000research.123041.2 (PMC9627402; doi:10.12688/f1000research.123041.2)
Supplement: Peer review history

# Peer review: Reviewer response for version 2

**DOI:** 10.5256/f1000research.138284.r150927

**Published:** 2022-10-31

**Authors:** Israa Al-ani

**Affiliations:** 1 Faculty of Pharmacy, Al-Ahliyya Amman University, Amman, Jordan

No specific comments, the work was executed with a perfect methodology.

 The authors used references up to 2021 in the field.

 Minor comments:

 -What about the safety of the lipids used? 

 -The system is to be used on the skin, what about the rheology of the system? spreadability? viscosity?

 Many thanks and good luck,

Is the work clearly and accurately presented and does it cite the current literature?

Yes

If applicable, is the statistical analysis and its interpretation appropriate?

Yes

Are all the source data underlying the results available to ensure full reproducibility?

Yes

Is the study design appropriate and is the work technically sound?

Yes

Are the conclusions drawn adequately supported by the results?

Yes

Are sufficient details of methods and analysis provided to allow replication by others?

Yes

Reviewer Expertise:

pharmaceutical formulation

I confirm that I have read this submission and believe that I have an appropriate level of expertise to confirm that it is of an acceptable scientific standard.

# Peer review: Reviewer response for version 1

**DOI:** 10.5256/f1000research.135104.r145632

**Published:** 2022-10-28

**Authors:** Ram Sahu

**Affiliations:** 1 Department of Pharmaceutical Sciences, Assam University, Silchar, India

Author inputs for the Vemurafenib formulation in the form of VEM-lipid complex are appreciable and novel work. The author successfully resolves the conventional issues related to the present drug by formulating into a new dosage form. The findings of VEM-lipid complex demonstrated highly improved the anticancer activity of the drug, as well as enhanced skin permeation and deposition with longer retention of the drug in a solubilized form in the skin dermis.

 But apart from this, I suggest the author make slight changes in the conclusion and reference section.

 Conclusion: The conclusion is lacking the issues of Vemurafenib for which this research was conducted and also elaborates the research findings, while other texts are fine. 

 References: Replacing the older references which are below than 2014 years with recent references.

Is the work clearly and accurately presented and does it cite the current literature?

Partly

If applicable, is the statistical analysis and its interpretation appropriate?

Yes

Are all the source data underlying the results available to ensure full reproducibility?

Yes

Is the study design appropriate and is the work technically sound?

Yes

Are the conclusions drawn adequately supported by the results?

Partly

Are sufficient details of methods and analysis provided to allow replication by others?

Yes

Reviewer Expertise:

My research area is novel drug delivery system

I confirm that I have read this submission and believe that I have an appropriate level of expertise to confirm that it is of an acceptable scientific standard.
